# Resistance to *Plum Pox Virus* (PPV) in apricot (*Prunus armeniaca* L.) is associated with down-regulation of two *MATHd* genes

**DOI:** 10.1186/s12870-018-1237-1

**Published:** 2018-01-27

**Authors:** Elena Zuriaga, Carlos Romero, Jose Miguel Blanca, Maria Luisa Badenes

**Affiliations:** 1 Citriculture and Plant Production Center, Instituto Valenciano de Investigaciones Agrarias (IVIA), CV-315, Km. 10.7, Moncada, 46113 Valencia, Spain; 2 Instituto de Biología Molecular y Celular de Plantas (IBMCP), Universidad Politécnica de Valencia-Consejo Superior de Investigaciones Científicas, Ingeniero Fausto Elio, s/n 46022, Valencia, Spain; 3 Instituto de Conservación y Mejora de la Agrodiversidad Valenciana (COMAV), Universidad Politécnica de Valencia, Ingeniero Fausto Elio, s/n 46022, Valencia, Spain

**Keywords:** *Plum Pox* virus, *Potyvirus*, MATHd, Silencing, PPV resistance, *Prunus*, Apricot

## Abstract

**Background:**

*Plum pox* virus (PPV), causing Sharka disease, is one of the main limiting factors for *Prunus* production worldwide. In apricot (*Prunus armeniaca* L.) the major PPV resistance locus (*PPVres*), comprising ~ 196 kb, has been mapped to the upper part of linkage group 1. Within the *PPVres*, 68 genomic variants linked in coupling to PPV resistance were identified within 23 predicted transcripts according to peach genome annotation. Taking into account the predicted functions inferred from sequence homology, some members of a cluster of meprin and TRAF-C homology domain (MATHd)-containing genes were pointed as PPV resistance candidate genes.

**Results:**

Here, we have characterized the global apricot transcriptome response to PPV-D infection identifying six *PPVres* locus genes (*ParP-1* to *ParP-6*) differentially expressed in resistant/susceptible cultivars. Two of them (*ParP-3* and *ParP-4*), that encode MATHd proteins, appear clearly down-regulated in resistant cultivars, as confirmed by qRT-PCR. Concurrently, variant calling was performed using whole-genome sequencing data of 24 apricot cultivars (10 PPV-resistant and 14 PPV-susceptible) and 2 wild relatives (PPV-susceptible). *ParP-3* and *ParP-4*, named as ***P****runus* ***ar****meniaca* ***P****PVres* **M**ATHd-**c**ontaining genes (*ParPMC*), are the only 2 genes having allelic variants linked in coupling to PPV resistance. *ParPMC1* has 1 nsSNP, while *ParPMC2* has 15 variants, including a 5-bp deletion within the second exon that produces a frameshift mutation. *ParPMC1* and *ParPMC2* are adjacent and highly homologous (87.5% identity) suggesting they are paralogs originated from a tandem duplication. Cultivars carrying the *ParPMC2* resistant (mutated) allele show lack of expression in both *ParPMC2* and especially *ParPMC1*.

**Conclusions:**

Accordingly, we hypothesize that *ParPMC2* is a pseudogene that mediates down-regulation of its functional paralog *ParPMC1* by silencing. As a whole, results strongly support *ParPMC1* and/or *ParPMC2* as host susceptibility genes required for PPV infection which silencing may confer PPV resistance trait. This finding may facilitate resistance breeding by marker-assisted selection and pave the way for gene edition approaches in *Prunus*.

**Electronic supplementary material:**

The online version of this article (10.1186/s12870-018-1237-1) contains supplementary material, which is available to authorized users.

## Background

Sharka disease, caused by *Plum pox* virus (PPV), is currently the most important viral disease affecting *Prunus* species [17]. PPV is a member of the *Potyvirus* genus in the *Potyviridae*, one of the largest families of plant viruses, and has been included in the ‘Top 10’ ranking of scientific/economically relevant plant viruses [46]. Described for the first time infecting plums (*Prunus domestica* L.) in Bulgaria around 1917 [3], PPV spread into most temperate fruit crop-growing areas since then [6]. The growth of PPV-resistant *Prunus* cultivars is pointed out as the ideal long-term solution, especially in endemic areas where fruit trees cannot be efficiently protected from Sharka infection [17]. However, resistant sources are scarce. Germplasm screenings have just identified a handful of North American apricot (*Prunus armeniaca* L.) PPV resistant cultivars [36] currently used as donors in the breeding programs, and a few plum genotypes showing tolerance or hypersensitive response to PPV infection [21].

Genetic control of PPV resistance in apricot has long been a source of controversy. Differences in the phenotyping methods and the use of distinct PPV strains hampered to reach firm conclusions [31]. However, most studies currently support the involvement of one major dominant locus (*PPVres* locus) located in the upper part of the apricot linkage group 1, including genetic mapping [15, 25, 26, 34, 41, 50] and genome-wide association approaches [35]. In a previous work, we narrowed down the *PPVres* locus to ~ 196 kb according to the peach genome syntenic region [58]. This study also pointed out a cluster of meprin and TRAF-C homology domain (MATHd)-containing genes as responsible for PPV resistance. Genetic evidence primarily supported the 5-bp insertion mutation disrupting one of these genes as the candidate variant causing resistance. Thus, since *PPVres* heterozygous genotypes confer resistance through the mutated *PPVres* allele, a gain-of-function or a dominant negative mutation was hypothesized [58]. In *Arabidopsis thaliana*, another MATHd-only protein-encoding gene, *RTM3*, is one of the dominant *RTM* genes involved in the restriction of PPV long distance movement [10]. In addition, the non-functionality of one or more *RTM* alleles is sufficient to abolish the resistance phenotype [10, 40]. On the contrary, PPV resistance in apricot is suggested to be associated with a *MATHd* mutated allele encoding a truncated non-functional protein [58]. Similarly, loss-of-function of the host eukaryotic translation initiation factor 4E isoform (*eIF(iso)4E*) has been shown to confer PPV resistance in *A. thaliana* and plum [14, 56]. However, resistance in these cases is due to ‘recessive homozygosity’ while, in apricot, natural PPV resistance is present in heterozygosis [58].

Genetic engineering technologies have been explored to obtain PPV resistant *Prunus* cultivars and rootstocks overcoming breeding limitations such as incompatibility barriers and long generation periods. Much of the work has involved pathogen derived resistance using constructs of the PPV coat protein (CP) gene (reviewed by [23]). This strategy was successfully implemented to develop the PPV resistant ‘HoneySweet’ C5 plum by RNA silencing activation against the PPV CP coding sequence ([47]; Patent No. US PP15154 P2). Host-derived resistance also has been exploited by silencing the host susceptibility (S) gene *eIF(iso)4E* to obtain PPV resistant transgenic plums [56]. However, transformation and regeneration in *Prunus* still are limiting factors for gene transfer technologies highly genotype-dependent [23]. No genome-edited *Prunus* species has been reported to date, but this technology will presumably have a great impact in PPV resistance breeding.

In this work we combined transcriptomic and genomic data to validate candidate genes previously identified by Zuriaga et al. [58] and to characterize the global transcriptome response to PPV infection in apricot. As a whole, results allowed us to support that PPV resistance relies on two down-regulated *PPVres* locus *MATHd* genes and to propose a dominant gene action via gene-silencing, both findings being relevant for future breeding in *Prunus*.

## Results

### Transcriptome profiles differ between resistant/susceptible apricot cultivars

To analyze the apricot response to PPV infection, PPV resistant cultivars ‘Goldrich’ and ‘Stella’ and the susceptible ‘Canino’ were grafted onto susceptible GF-305 peach rootstocks, and half of the plants were inoculated by chip budding with PPV-D. Gene expression was analyzed in a total of 16 leaf RNA samples using pair-ends (PE) HiSeq2000 Illumina sequencing. More than 490 M of 101 bp raw sequence reads were obtained, averaging 30.63 M per sample (Additional file 1: Table S1). After quality trimming and adapter clipping, a total of ~ 48,885 M of high quality sequenced bases (99.75% of raw sequenced bases) belonging to 70–100 bp cleaned reads were obtained. As suggested by Haas et al. [19], normalized cleaned sequences were assembled by Trinity software. After refinement (see Experimental procedures section for details), 91,735 transcripts were generated and grouped into 61,096 genes, with 98,391,234 bases and a contig mean length of 1072.56 bases. Regarding other *Prunus* species, comparable results were obtained studying the response to PPV infection in plum [44], the response of reproductive tissues to frost stress in almond (*Prunus dulcis* (*Mill*.) *D.A. Webb*) [37], and the dynamics of fruit development [1] and the anthocyanin biosynthesis in sweet cherry (*Prunus avium* L.) [57]. *Prunus persica* reference transcriptome (peach v.1.0, rosaceae.org), obtained from different tissues (i.e. fruits, roots, leaves, embryos and cotyledons) has 28,689 transcripts [54]. Up to 10,894 peach transcripts are represented in this leaf RNA only-based apricot transcriptome with a length coverage over 80% (Additional file 2: Table S2). Putative orthologs were detected in peach for 34% of the total apricot assembled transcripts by using the reciprocal best Blast hit (RBH) criterion (Additional file 3: Data S1).

*Plum pox virus* genome sequence (NCBI Reference Sequence: NC_001445.1) was blasted (e-value >1e-07) against the apricot assembled contigs and just one contig (*c34934_g0_i1*) showed similarity with the virus sequence (Additional file 4: Table S3). PPV genome was almost completely assembled into this single contig, with 9741 bases length and more than 98% identity, except for a small portion of both ends and a 45 bases insertion. Reads mapping against this ‘PPV contig’ were used to check the presence of the virus (Additional file 5: Table S4). As expected, PPV was significantly present in the 3 replicates of the PPV-inoculated susceptible ‘Canino’ but almost absent in the rest. PPV symptoms were clearly observed in all inoculated GF305 rootstock indicators.

Transcript abundance estimates for each sample were obtained by aligning trimmed PE reads to the assembled transcriptome. In order to identify trends or detect putative biases in the data set, relationships between samples were checked using multidimensional scaling (MDS) plots (Additional file 6: Figure S1). As a whole, transcriptome profile variability was higher between cultivars than it was between infection conditions (I: inoculated; NI: non-inoculated) (Additional file 6: Figure S1a). PPV-inoculated ‘Goldrich’ replicate ‘Go_I_rep3’ was clearly separated from the rest of the ‘Goldrich’ samples and therefore it was eliminated for subsequent analyses to prevent background noise. Within cultivar, ‘Canino’ (Additional file 6: Figure S1b) and ‘Stella’ (Additional file 6: Figure S1d) samples appear separated according to the infection conditions but not in ‘Goldrich’ (Additional file 6: Figure S1c). Technical replicate pairs (‘Ca_NI_rep2a’/'Ca_NI_rep2b’; ‘Go_I_rep1a’/'Go_I_rep1b’; ‘Go_I_rep2a’/'Go_I_rep2b’) cluster together respectively and were considered as single biological replicates (‘Ca_NI_rep2’, ‘Go_I_rep1’, and ‘Go_I_rep2’) for subsequent analyses.

**Fig. 1:**
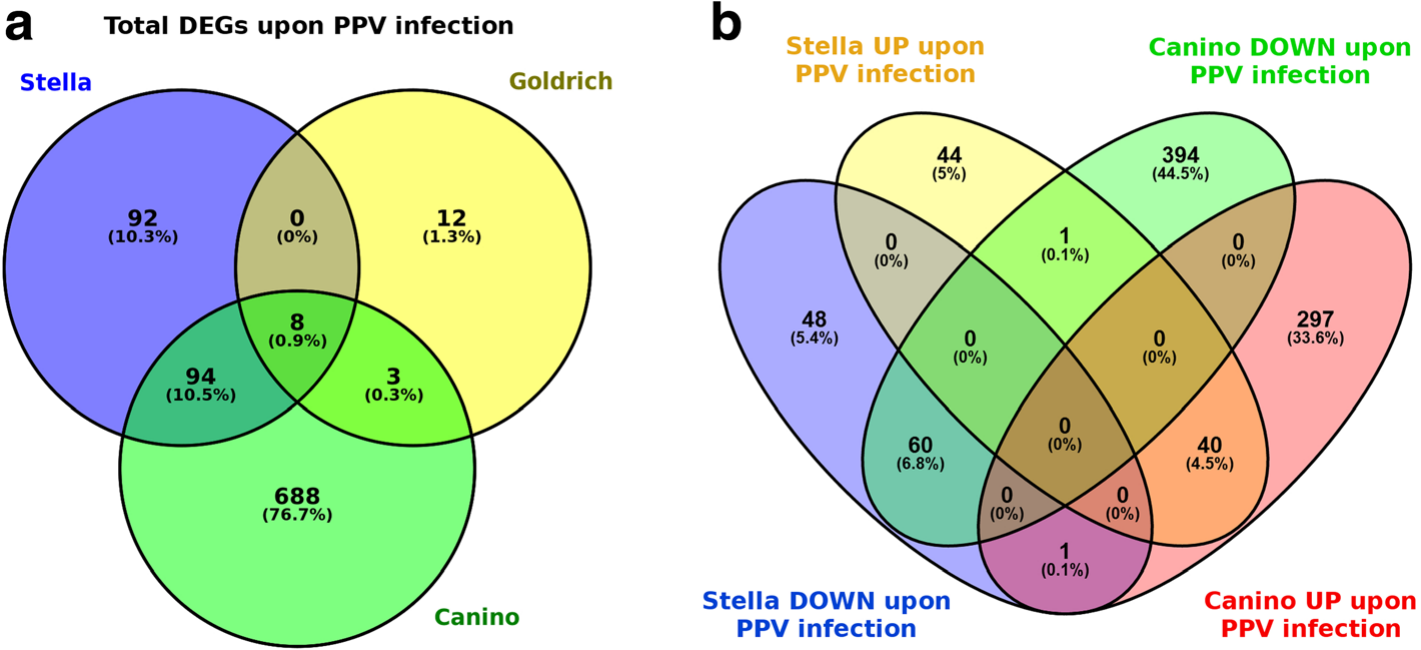
Venn diagrams showing the number of DEGs identified comparing PPV inoculated and non-inoculated plants for each cultivar. **a** Total number of DEGs. **b** Numbers of up- and down-regulated genes upon PPV infection in ‘Canino’ and ‘Stella’ cultivars

Number of differentially expressed genes (DEGs) between I and NI infection conditions differs for each cultivar: 793 in ‘Canino’ (homozygous for PPV susceptibility), 194 in ‘Stella’ (homozygous for PPV resistance) and 23 in ‘Goldrich’ (heterozygous) (Fig. 1a). ‘Canino’ and ‘Stella’ share in common 102 DEGs while 688 appear exclusively in ‘Canino’ and 92 in ‘Stella’. Two common DE cell-wall related genes exhibit opposed behaviors in both cultivars, *c29444_g0* appears down-regulated in inoculated ‘Canino’ and over-expressed in inoculated ‘Stella’, while the opposite occurs with *c33041_g0* (Fig. 1b). To gain insights into the biological roles of DEGs in both cultivars, a Gene Ontology (GO) categories enrichment analysis (Fisher exact test, with a FDR < 0.05) was performed using Blast2GO (Table 1). In ‘Canino’, we found GO terms for molecular functions significantly enriched in binding (iron ion, chromatin and DNA) and catalytic activities (oxidoreductase, hydrolase, lyase, transferase and transporter). For biological processes, enriched GO terms were related to abiotic stimulus response. Finally, for cellular component the enriched GO terms were related to chloroplasts (thylakoids and stroma), nucleus and nucleosome. In ‘Stella’ we only found enriched GO terms for biological processes related to the response to abiotic and endogenous stimulus, circadian rhythm and positive development regulation.

**Table 1:** GO categories enrichment analysis (Fisher exact test) of differentially expressed genes (DEGs) identified within cultivar against PPV infection using Blast2GO. Cultivar, Category (MF: Molecular Function, BP: Biological Process, CC: Cellular Component), GO-ID, Term, FDR (False Discovery Rate) and *p*-value are indicated

Cultivar	GO Category	GO ID	GO Name	FDR	P-Value	N. Genes
Canino	BIOLOGICAL PROCESS	GO:0009414	response to water deprivation	0,0002	3,39E-007	26
		GO:0009637	response to blue light	0,0051	2,16E-005	11
	CELLULAR COMPONENT	GO:0031977	thylakoid lumen	0,0012	3,39E-006	11
		GO:0009570	chloroplast stroma	0,0179	9,26E-005	36
		GO:0048555	generative cell nucleus	0,0402	2,97E-004	3
		GO:0000786	Nucleosome	0,0404	3,05E-004	7
		GO:0009534	chloroplast thylakoid	0,0407	3,18E-004	24
	MOLECULAR FUNCTION	GO:0016491	oxidoreductase activity	0,0007	1,79E-006	100
		GO:0005506	iron ion binding	0,0031	1,11E-005	29
		GO:0003682	chromatin binding	0,0223	1,27E-004	21
		GO:0016161	beta-amylase activity	0,0362	2,35E-004	4
		GO:0003677	DNA binding	0,0402	2,82E-004	75
		GO:0008792	arginine decarboxylase activity	0,0402	2,97E-004	3
		GO:0004645	phosphorylase activity	0,0407	3,22E-004	4
		GO:0016760	cellulose synthase (UDP-forming) activity	0,0409	3,27E-004	8
		GO:1,901,505	carbohydrate derivative transporter activity	0,0457	3,99E-004	8
Stella	BIOLOGICAL PROCESS	GO:0009644	response to high light intensity	0,0024	2,43E-007	9
		GO:0048582	positive regulation of post-embryonic development	0,0071	1,45E-006	6
		GO:0009408	response to heat	0,0411	3,42E-005	9
		GO:0007623	circadian rhythm	0,0411	3,96E-005	6
		GO:0009719	response to endogenous stimulus	0,0411	4,58E-005	21

### Two *PPVres* locus *MATHd* genes are down-regulated in apricot resistant cultivars

**Fig. 2:**
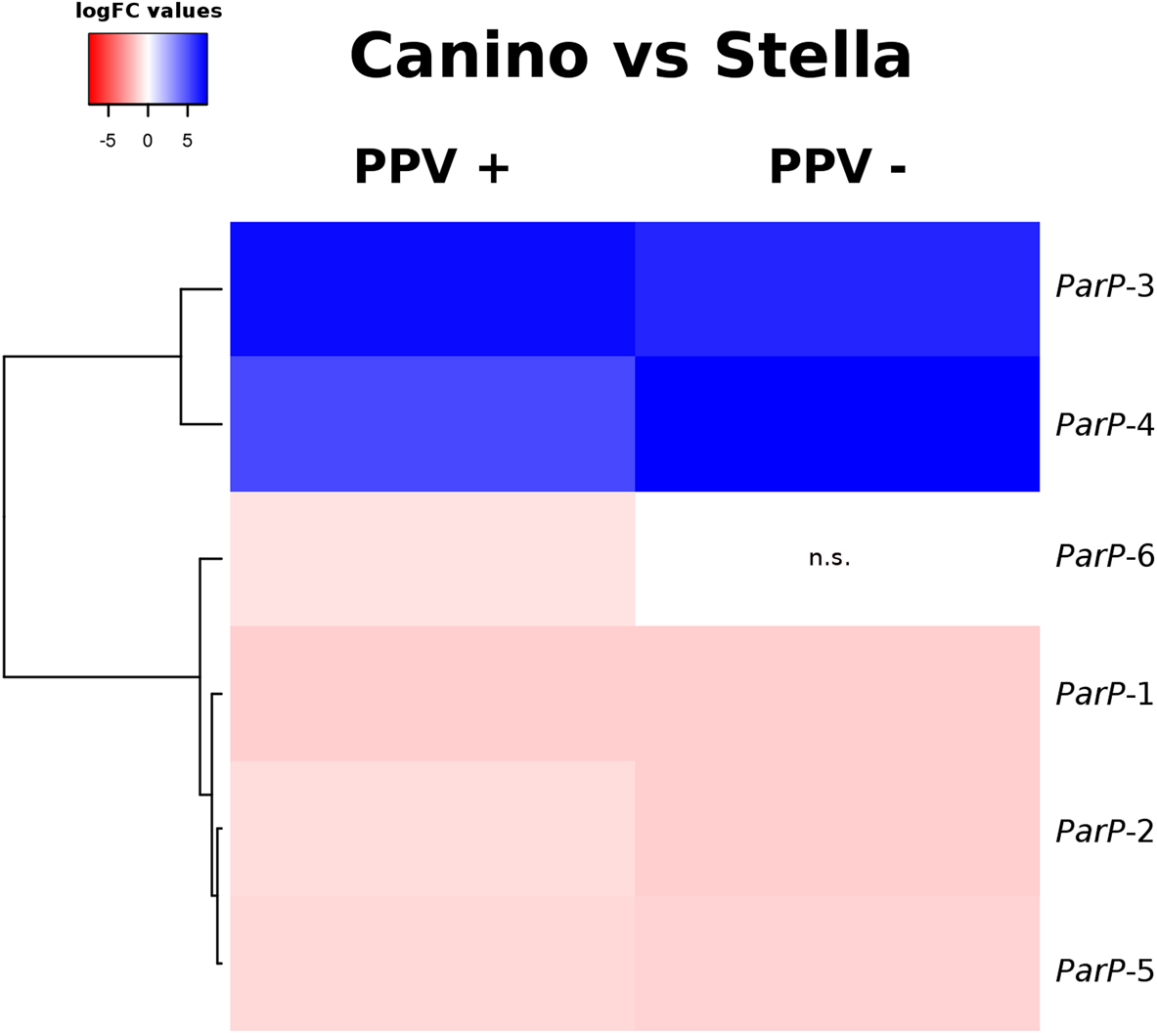
Heat map of RNA-seq expression levels for the identified *PPVres* locus DEGs between the resistant ‘Stella’ and the susceptible ‘Canino’ cultivars. Blue positive log fold-change (logFC) indicates higher expression in the cultivar ‘Canino’ than in ‘Stella’. Columns represent comparison between PPV inoculated (PPV+) and non-inoculated (PPV-) samples, respectively. The gene clustering is drawn on the left. Non-significant differences with *p*-values > 0.05 are indicated (n.s.)

**Fig. 3:**
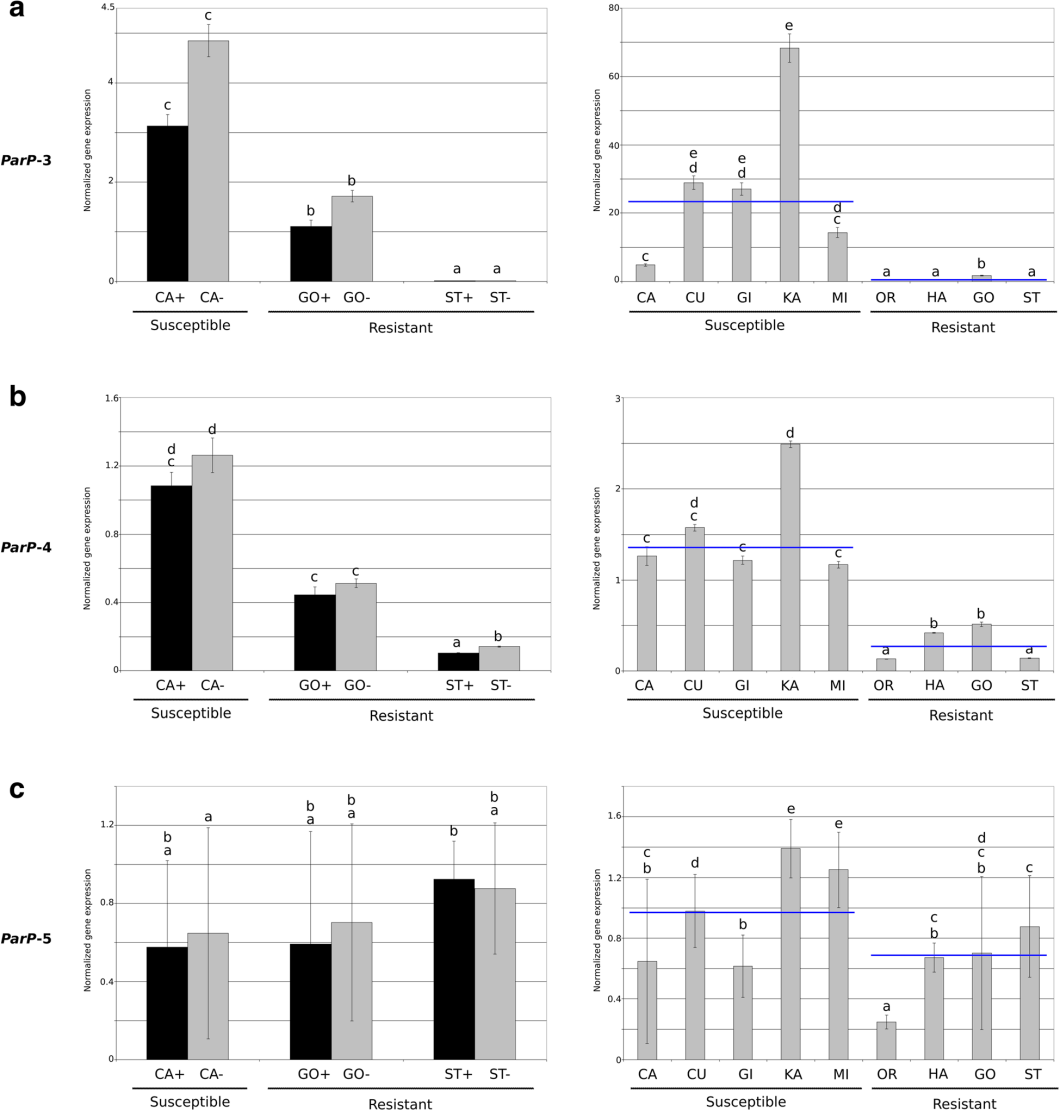
qRT-PCR analysis of *PPVres* locus *MATHd* genes showing differential expression according to RNA-seq data. **a** *ParP-3*. **b** *ParP-4*. **c** *ParP-5*. Normalized expression levels were obtained using the housekeeping genes *Actin* and *Sand-like* as controls. Data are means from 1 to 3 biological samples with three technical replicates for each one. Error bars represent standard deviation and different letters indicate significant differences (*P* < 0.05). Left: Histograms of gene-expression using PPV-inoculated (+) and non-inoculated (−) plants of ‘Canino’ (CA), ‘Goldrich’ (GO) and ‘Stella’ (ST). Right: Histograms of gene-expression of non-inoculated plants of 5 PPV susceptible (CA: ‘Canino’, CU: ‘Currot’, GI: ‘Ginesta’, KA: ‘Katy’, MI: ‘Mitger’) and 4 PPV resistant (OR: ‘Orange Red’, HA: ‘Harlayne’, GO: ‘Goldrich’, ST: ‘Stella’) cultivars. Blue lines indicate mean value obtained after removing extreme values

The major dominant *PPVres* locus in apricot comprises ~ 196 kb according to the peach genomic syntenic region [58]. Nineteen assembled apricot genes were RBH and other ten showed highly similarity with some peach annotated genes within the *PPVres* locus (Additional file 7: Table S5). None of them was DE within cultivar comparing PPV infection conditions but 6 (*ParP*-1 to *ParP-6*) appear significantly DE between cultivars (Fig. 2, Additional file 8: Table S6). *ParP-3* and *-4* were highly down-regulated in ‘Stella’ (absolute logFC values between 5.19–7.4), while *ParP-1*, − *2*, *− 5* and *− 6* were up-regulated (absolute logFC values between 0.78–1.45). *ParP-3*, *ParP-4* and *ParP-5* are homologous to 3 peach *MATHd* genes (*ppa022254m*, *ppb0221 95 m* and *ppa008951m*, respectively) previously pointed as PPV resistance candidate genes [58]. RNAseq results were further confirmed by qRT-PCR analyses that revealed similar gene-expression patterns (Fig. 3). For instance, no statistically significant differences (*p* < 0.05) were detected within cultivars under different infection conditions. *ParP-3* and *ParP-4* were clearly down-regulated in ‘Stella’ with regard to ‘Canino’, while ‘Goldrich’ occupied an intermediate position between them (Fig. 3a-b). *ParP-3* showed the highest relative expression differences, ~ 300-fold increase in non-inoculated ‘Canino’ compared with ‘Stella’ (Fig. 3a, Additional file 9: Table S7). On the contrary, *ParP-5* showed no significant differences neither between nor within samples (Fig. 3c). Correlation between these gene-expression patterns and the PPV-resistance phenotype was confirmed by testing two additional resistant cultivars (‘Harlayne’ and ‘Orange Red’, both *PPVres* heterozygous) and four susceptible cultivars (‘Currot’, ‘Ginesta’, ‘Katy’ and ‘Mitger’) (Fig. 3 ; Additional file 9: Table S7). *ParP-3* and *ParP-4* were found clearly down-regulated in all resistant cultivars (Fig. 3a-b), and *ParP-3* again showed more striking differences ranging between ~ 300 to ~ 4267-fold higher gene-expression in susceptible cultivars with regard to ‘Stella’ (Additional file 9: Table S7). Moreover, *ParP-5* did not show consistent differences between susceptible and resistant cultivars (Fig. 3c).

### *ParP-3* and *ParP-4* accumulate genomic variants linked to PPV resistance

**Fig. 4:**
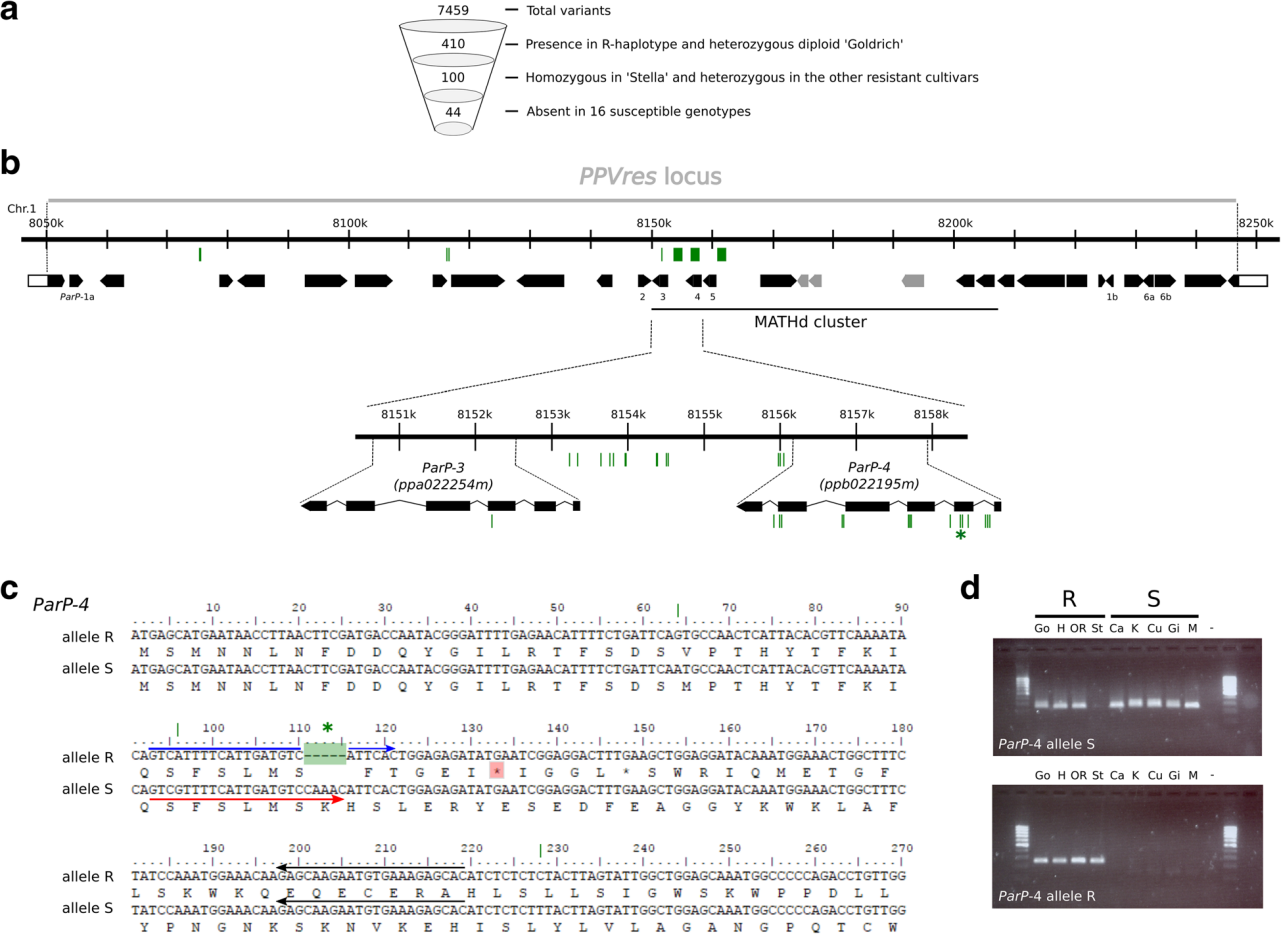
Identification of the *PPVres* locus variants mediating PPV resistance in apricot. **a** Variant filtering of SNPs and small INDELs within the *PPVres* locus called using 24 apricot cultivars and 2 apricot relatives WGS. **b** Positions of filtered variants in the peach syntenic region (*green lines*) corresponding to the *PPVres* locus. *MATHd* genes cluster is indicated and peach *MATHd* genes absent in the apricot genome appear *grey colored*. Variants in *ParP-3* and *ParP-4* (putative orthologs of *ppa022254m* and *ppb0221 95 m*) are detailed below. The 5-bp deletion causing a frameshift mutation is labeled with an asterisk. **c** *ParP-4* CDS and predicted amino acid sequences for the resistant (R) and susceptible (S) alleles. The 5-bp deletion (*green boxed*) leads to a premature stop-codon *(red boxed*) in the R-allele. qRT-PCR primer positions were indicated by arrows (*blue*, forward R-allele-specific; *red*, forward S-allele-specific; *black*, reverse). (d) *ParP-4* allele-specific PCR-genotyping in 4 PPV resistant and 5 PPV susceptible apricot cultivars (GO: ‘Goldrich’; HA: ‘Harlayne’; OR: ‘Orange Red’; ST: ‘Stella’; CA: ‘Canino’; KA: ‘Katy’; CU: ‘Currot’; GI: ‘Ginesta’; MI: ‘Mitger’)

Whole genome sequences (WGS) of 24 apricot cultivars (10 PPV-resistant and 14 PPV-susceptible) and two apricot relatives (PPV-susceptible), as well as reads derived from six BAC clones corresponding to the ‘Goldrich’ *PPVres* locus R-haplotype, were aligned against the peach genome (peach v1.0; http://www.rosaceae.org) (Additional file 10: Table S8). Variant calling to detect SNPs or small insertions and deletions (INDELs) within the *PPVres* locus identified 2424 to 3928 putative SNPs and 425 to 711 putative INDELs per genotype (Additional file 10: Table S8). Afterwards, three filters were sequentially applied to discriminate SNPs/INDELs associated with PPV resistance from all 7459 detected variants: i) variants should be linked in coupling with PPV-resistance, as confirmed by their presence in ‘Goldrich’ R-haplotype BACs, being heterozygous in diploid ‘Goldrich’ WGS; ii) they should be homozygous in ‘Stella’ and heterozygous in the rest of the resistant cultivars; iii) they had to be absent in all 16 susceptible cultivars (Fig. 4a, Additional file 11: Table S9). A total of 44 SNP/INDELs fulfilled these three conditions. Twenty-eight of these variants were found in intergenic regions, being 14 and 11 in the putative promoter regions of *ParP-3* and *ParP-4*, respectively (Fig. 4b). In addition, 1 filtered variant was present within *ParP-3* (nsSNP: Ile109Leu) and 15 within *ParP-4* (7 in intronic regions, 5 sSNPs, 2 nsSNPs: Glu194Lys and Thr266Ala, and 1 5-bp deletion) (Fig. 4b). Interestingly, this latter 5-bp deletion is located in the second exon and produces a frameshift mutation that creates a premature stop codon (Fig. 4c). The 5-bp deletion could be consistently screened on agarose gel electrophoresis by allele-specific PCR in susceptible and resistant cultivars providing a useful tool for marker-assisted selection (MAS) into apricot breeding programs (Fig. 4c-d). Following the gene naming guidelines of the Genome Database for Rosaceae (GDR) [24], *ParP-3* and *-4* were respectively named *ParPMC1 and ParPMC2* (*Prunus* *armeniaca* *PPVres* locus MATH-domain containing (*PMC*) genes).

**Fig. 5:**
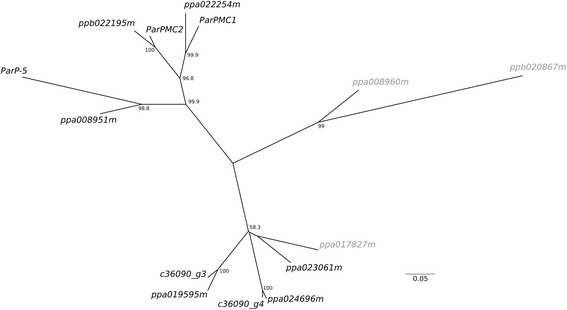
Maximum Likelihood phylogenetic tree of peach and apricot *MATHd* genes clustered in the *PPVres* locus. Confident positions (935 bases) from the alignment of CDS sequences were used. The Tamura 3-parameter model (T92) + G was used as the best-fitting evolutionary model. Bootstrapping support values of the nodes > 50 (using 500 replications) are indicated

Five apricot genes were identified as putative orthologs or highly similar to the 9 peach *PPVres* locus *MATHd* genes according to reciprocal Blast results (Additional file 7: Table S5). Maximum likelihood based phylogeny of these 14 genes revealed 3 highly supported sub-clusters (Fig. 5). *ParPMC1*, *ParPMC2* and *ParP-5*, and their putative peach orthologs, *ppa022254m*, *ppb0221 95 m* and *ppa008951m,* grouped together in the same sub-cluster. *ParPMC1* and *ParPMC2* showed the shortest genetic distance among all apricot pairs (0,148) having 87,5% of sequence identity (Additional file 12: Table S10).

## Discussion

### Apricot response against PPV infection

Differences in transcriptome profiles were more striking between apricot cultivars than between viral infection conditions. Different factors related or not with PPV infection may explain this. For instance, ‘Goldrich’ and ‘Stella’ are North American apricot cultivars adapted to cold-growing conditions while the Spanish ‘Canino’ is mainly grown through the temperate Mediterranean Basin [29]. PPV inoculation procedure entails a cold treatment to break dormancy [38]. Accordingly, distinct cultivar-dependent responses to this treatment could be expected as reflected by their global gene-expression profiles. As expected, deeper differences in transcriptome profiles between PPV inoculated and non-inoculated tissues were observed in the susceptible materials. In accordance with previous works, this expression-pattern might be due to changes suffered by cells experiencing pathogenic stress. Taking into account the broad differences among previous studies, similar biological processes and molecular functions were found to be affected in this work. In apricot, Rubio et al. [45] identified DEGs involved in biological processes associated with responses to different stimuli. Wang et al. [55] observed up-regulation of genes involved in defense, cellular transport, development, protein synthesis and binding functions in peach infected leaves. Studies on the response to PPV infection in *Arabidopsis* leaves identified altered genes belonging to major groups of metabolism, transcription/splicing/RNA processing proteins, defense and development/storage proteins [4]. Enriched GO terms for cellular components are in agreement with papers describing the effect of PPV on the photosynthetic processes producing physical and biochemical changes in the chloroplasts [11, 22, 44, 49]. As a whole, transcriptomic data provided in this work have sharpened our knowledge on the response to PPV at gene-expression level and might be helpful to search for additional genes involved in PPV resistance.

### PPV resistance in apricot is mediated by *MATHd* gene/s

The *PPVres* locus syntenic region in peach contains 29 genes (one of them encoding for 3 alternative transcripts) as annotated by the International Peach Genome Initiative. Gene-expression analysis in this work showed that none of the corresponding apricot genes was DE between infected/non-infected tissues, suggesting that PPV-D presence does not modulate their expression. However, six DEGs were identified between the susceptible ‘Canino’ and the resistant ‘Stella’. Two of them, *ParPMC1* and *ParPMC2*, were significantly DE emerging as the best candidates from expression data. Previously, using genomic data of three PPV-resistant and four PPV-susceptible cultivars, a total of 68 variants linked in coupling with PPV resistance were identified in 23 apricot genes located at the ~ 196 kb *PPVres* locus [58]. In this work, a much deeper variant calling analysis based on 26 apricot WGS, allowed us to confirm 44 variants matching with the imposed criteria by the genetics of PPV resistance in apricot [50, 53]. *ParPMC1* and *ParPMC2* genes contain 1 and 15 of these variants, respectively, while another 14 and 11 are located in their putative promoter regions. Remarkably, both genes are homologous to members of the peach *MATHd* genes cluster previously suggested as the most likely PPV resistance candidate genes [58]. *ParPMC1* has only one nsSNP (Ile109Leu) within the first MATH domain but *ParPMC2* accumulates 15 variants including a 5 nt deletion that results in a premature stop codon. *ParPMC1* and *ParPMC2* are highly similar (87.5% identity), close to *ParP-5*, and all 3 occupy adjacent positions in the *MATHd* genes cluster. Therefore, we hypothesize that they are paralogs originated by tandem duplications from a single ancestral gene. However, their expression patterns are different, *ParPMC1* and *ParPMC2* are down-regulated in PPV resistant cultivars but *ParP-5* expression seems to be similar in both PPV resistant and susceptible cultivars. *ParPMC1* remains basically unaltered while the *ParPMC2* resistant allele has accumulated deleterious mutations probably as a consequence of a pseudogenization process [16]. It could be speculated that this, in turn, affects the expression of *ParPMC1* and *ParPMC2* susceptible alleles but not *ParP-5* expression due to sequence differences. Thus, it may also be suggested that *ParP-5* function is not redundant to *ParPMC1* and *ParPMC2*. Altogether, these results support that *ParPMC1* and/or *ParPMC2* genes are required for PPV susceptibility in apricot. Other loci contributing to PPV resistance have been proposed in studies using PPV-Rec [34] and PPV-M strains [35], but consistent candidate genes remain to be identified. Moreover, Decroocq et al. [12, 13] observed that some apricot genotypes carrying the *ParPMC2* resistant allele are susceptible to PPV, mainly to the M strain. These reports suggest that *ParPMC2* resistant allele does not suffice to confer PPV resistance being necessary additional genes. This point cannot be discarded on the basis of the evidence provided in this work. However, all available studies (including these latter) undoubtedly show a tight linkage between *ParPMC2* resistant allele and PPV-D resistance. Moreover, misfits may be due to other genes but also to the PPV strain (different genes may underlie PPV-M resistance).

### Down-regulation of *MATHd* gene/s expression causes PPV resistance in apricot

Down-regulation of *ParPMC1* and *ParPMC2* genes expression is the differential factor between PPV resistant and susceptible apricot cultivars regarding the *PPVres* locus. Mutations accumulated in the promoter regions of the PPV resistant alleles may be directly affecting their expression, but half-gene dosage can not account for the observed differences between resistant and susceptible cultivars. Nevertheless, *ParPMC1* and *ParPMC2* gene expression may also be prevented by gene-silencing. For instance, nonsense-mediated mRNA decay (NMD) is a conserved mechanism that targets aberrant mRNAs carrying premature termination codons for destruction, preventing the accumulation of potentially harmful truncated proteins [33]. According to these authors, a typical target has a termination codon positioned more than 50–55 nt upstream of the last exon-exon junction or has a long 3′ untranslated region. Interestingly, *ParPMC2* has a 5-bp loss-of-function deletion in the second exon, 73 nt upstream of an exon–exon junction, being a potential target for NMD. Pseudogenes are suggested to potentially regulate their protein-coding cousins [42], as exemplified by the silencing of a gene required for sexual seed formation in apomictic *Paspalum simplex* associated with its homolog pseudogene [48]. Moreover, overlaps between RNA silencing and NMD pathways have been reported in plants. Christie et al., [8] pointed out that when aberrant mRNA formation is frequent (i.e. due to nonsense mutations) there is a threshold-dependent induction of RNA silencing. According to gene-expression data, apricot cultivars carrying only one *ParPMC2* resistant (mutated) allele showed lack of expression of both *ParPMC2* and *ParPMC1* genes, but especially *ParPMC1*. This scenario is consistent with a dominant mutation in *ParPMC2* conferring PPV resistance by silencing functional homologs such as the susceptible (non-mutated) *ParPMC2* allele and/or *ParPMC1*. Therefore, *ParPMC1* and *ParPMC2* can be considered host susceptibility (recessive) genes which silencing may confer PPV resistance trait.

## Conclusions

Together with the eIF(iso)4E–like factor [56], *ParPMC1* and *ParPMC2* are, to our knowledge, the first factors required for PPV susceptibility identified in *Prunus*, the natural hosts of PPV. Functional analyses are currently in progress to elucidate the exact role of *ParPMC1* and *ParPMC2* genes in PPV resistance. Meanwhile, silencing of these and other recessive genes, such as the eIF(iso)4E–like factor, by genome editing technologies seems a promising strategy to exploit host-derived resistance in *Prunus* pyramiding durable resistance to PPV. Furthermore, closer analysis of other DEGs in the susceptible cultivar ‘Canino’ may allow the identification of additional host S-genes involved in viral infection.

## Methods

### Plant material and PPV inoculation

The PPV resistant cultivars ‘Goldrich’ and ‘Stella’ and the susceptible ‘Canino’ were used in the RNAseq experiment. These cultivars are maintained as part of the germplasm collection at IVIA (Valencia, Spain). Each genotype was grafted onto PPV susceptible ‘GF305’ peach seedlings growing in pots under controlled greenhouse conditions as described by Moustafa et al. [38]. Half of the plants were inoculated by chip budding on the GF305 rootstock using the PPV Dideron strain 3.3 RB [2]. Four weeks after grafting, plants were subjected to an artificial period of dormancy in darkness at 5 °C for 8 weeks. Subsequently, the plants were transferred again to the greenhouse. After 8 weeks, young and fully developed leaves, randomly distributed along the sprout, were harvested and directly frozen in liquid nitrogen in the greenhouse before RNA extraction. The presence of virus was scored by visual inspection of symptoms in susceptible ‘GF-305’ leaves and by detecting PPV transcripts through RNAseq analysis. Three replicates for each condition (inoculated/non-inoculated) were sampled from ‘Goldrich’ and ‘Canino’ and two from ‘Stella’.

Additionally, 2 resistant (‘Harlayne’ and ‘Orange Red’) and 5 susceptible (‘Canino’, ‘Currot’, ‘Ginesta’, ‘Katy’ and ‘Mitger’) cultivars were employed for gene expression analyses. All of them also maintained as part of the germplasm collection at IVIA (Valencia, Spain).

### Total RNA isolation and sequencing

Total RNA was isolated from 100 mg of powdered leaves with the RNeasy Plant Mini Kit (Qiagen, Valencia, CA, USA), adding 1% (w:v) polyvinylpyrrolidone (PVP-40) to the kit extraction buffer before use, followed by a DNase treatment with the RNase-Free DNase Set (Qiagen, Valencia, CA, USA). RNA quality and quantity were checked by agarose gel electrophoresis and Nanodrop ND-1000 spectrophotometer (Nanodrop Technologies, Wilminton, DE, USA). Library construction and sequencing were performed by the Beijing Genomics Institute (BGI Tech Solutions (Hong Kong) Co., LTD). RNA quality and quantity were rechecked before sequencing by BGI using both Agilent 2100 Bioanalyzer (Agilent Technologies, Santa Clara, CA, USA) and Nanodrop. ‘Short-insert’ libraries were sequenced using an Illumina HiSeq2000 instrument for PE sequencing of 101-bp. Some samples were sequenced twice to obtain the amount of clean data needed and treated as technical replicates in subsequent analyses. Cleaned paired-end sequence dataset was deposited in the NCBI Short Read Archive (SRA) under the accession numbers SRR5591366 to SRR5591375, associated with the BioProject PRJNA387702.

### De novo transcriptome assembly and quality control

FastQC v.0.10.1 (http://www.bioinformatics.babraham.ac.uk/projects/fastqc/) software was used to assess the quality of raw and clean read sets. Reads were quality trimmed using FASTX-toolkit (http://hannonlab.cshl.edu/fastx_toolkit) with a minimum quality score of 25 and a minimum length of 40. Adaptor sequences were trimmed using the ‘trim_blast_short’ script available as part of seq_crumbs (http://bioinf.comav.upv.es/seq_crumbs/). High quality reads of all samples were combined in order to perform the de novo assembly using the Trinity v.20140413p1 software [18] (see Additional file 13: Methods S1 for details).

In order to check the quality of the assembly, obtained transcripts were blasted against the GDR peach transcripts database v.1.0 (ftp://ftp.bioinfo.wsu.edu/species/Prunus_persica/Prunus_persica-genome.v1.0/genes/) for similarity searches (top 25 hits at e-value >1e-07). Results allowed us to estimate the number and length of the genes recovered using the ‘analyze_blastPlus_topHit_coverage.pl’ script from the Trinity software. Assembled transcriptome was deposited in the NCBI Transcriptome Shotgun Assembly Sequence (TSA) Database associated to the BioProject PRJNA387702.

### De novo transcriptome annotation

Gene ontology (GO) annotation was performed using the Blast2GO software [9] and the Blastx results against the NCBI non-redundant protein (nr) database (http://www.ncbi.nlm.nih.gov/protein). Blastx Reciprocal Best Hits (RBH) analysis was performed to obtain a set of putative orthologs between apricot and peach using peptides contained in the GDR peach v1.0 database. *Plum pox virus* genome sequence (NCBI Reference Sequence: NC_001445.1) was blasted (e-value >1e-07) against the apricot assembled transcripts in order to identify virus sequences. The ngs_backbone software [5] was employed for annotations from Blast results in all cases, except for the GO term annotation. Blast analyses were conducted using Picasso supercomputer from the Supercomputing and Bioinnovation Center at the University of Málaga (http://www.scbi.uma.es). Rest of the analyses were made using the Bioinformatics Department server (http://bioinf.comav.upv.es/) of the Instituto de Conservación y Mejora de la Agrodiversidad Valenciana (COMAV) at the Polytechnic University of Valencia.

### Differential expression analysis

Cleaned RNA-seq reads were aligned to the assembled transcriptome using Bowtie [28] through the Trinity software [19]. Transcript quantification was performed with RSEM [30] and the edgeR package [43] was used to call differentially expressed genes (DEGs). Samples considered as technical replicates were analyzed both independently and combined as a single sample. False discovery rate (FDR) ≤0.05 was used to determine the threshold of the *p*-value in multiple tests. Relations between samples were observed using multidimensional scaling (MDS) plots, where distance between each pair of samples can be interpreted as the leading log-fold change between the samples for the genes that best distinguish that pair of samples. By default, leading fold-change is defined as the root-mean-square of the largest 500 log2-fold changes between that pair of samples. In our case, MDS plots were obtained using those 500 genes and also using all genes, with no significant differences between both approaches. GO enrichment analysis of DEGs was performed using Blast2GO software with a cutoff value of FDR ≤ 0.05. Venn diagrams were obtained using the tool disposable in http://bioinfogp.cnb.csic.es/tools/venny/index.html. Heat-map was performed using a custom R script.

### qRT-PCR analysis

Four PPV resistant (‘Goldrich’, ‘Stella’, ‘Harlayne’ and ‘Orange Red’) and 5 PPV susceptible (‘Canino’, ‘Currot’, ‘Ginesta’, ‘Katy’ and ‘Mitger’) cultivars were analyzed by qRT-PCR. Total RNA (500 ng) was reverse transcribed with the PrimeScript RT reagent kit using an Oligo-d(T) primer (Takara Bio, Otsu, Japan) in a total volume of 10 μl. Two microliters of 10X diluted first-strand cDNA were used for PCR reactions in a final volume of 20 μl. qRT-PCR was performed on a StepOnePlus Real-Time PCR System (Life Technologies, Carlsbad, CA, USA), using SYBR premix Ex Taq (Tli RNaseH plus) (Takara Bio). Primer pairs are listed in Additional file 14: Table S11. Cycling protocol consisted of 10 min at 95 °C, followed by 40 cycles of 15 s at 95 °C for denaturation and 1 min at 60 °C for annealing and extension. PCR reaction specificity was assessed by the presence of a single peak in the dissociation curve after amplification and through size estimation of the amplified products by agarose electrophoresis. Normalized gene expression levels were measured by the relative standard curve procedure using the geometric mean of two reference genes, *Actin* and *Sand*-*like* [32]. Results were the average of 1–3 independent biological replicates with 3 technical replicates each one. Comparisons of multiple samples were evaluated by the non-parametric Kruskal-Wallis test, with a confidence level of 95%, using the Statgraphics Centurion XVII v. 17.2.00 software (Statpoint Technologies, Warrenton, VA, USA). Significantly different samples were labelled with different letters.

### WGS mapping, variant calling and filtering

WGS of 10 PPV resistant and 14 PPV susceptible cultivars and 2 PPV susceptible apricot relatives were used in this study (Additional file 10: Table S8). Ten of these WGS, and the 454 sequenced BAC clones belonging to the ‘Goldrich’ *PPVres* locus R-haplotype, were available from our previous works [39, 58]. Other 16 WGS were downloaded from the SRA repository (https://www.ncbi.nlm.nih.gov/sra). All raw reads were processed using the ‘run_trimmomatic_qual_trimming.pl’ script from the Trinity software. After removing the low-quality regions as well as vector and adaptor contaminants, cleaned reads were aligned to the peach genome v1.0 (ftp://ftp.bioinfo.wsu.edu/species/Prunus_persica/Prunus_persica-genome.v1.0/) using Bowtie2 v2.2.4 software [27]. Variant calling to detect SNPs or small INDELs was performed using HaplotypeCaller tool from the Genome Analysis Toolkit (GATK) v3.5–0-g36282e4 software [52] setting the minimum phred-scaled quality score of 10 in emission confidence and of 30 in calling confidence. Following the GATK Best Practices (https://software.broadinstitute.org/gatk/best-practices/), variant discovery analysis was performed as cohort of samples. In order to discriminate variants linked in coupling with PPV resistance from all the detected variants, 3 filters were sequentially applied using homemade python scripts: (i) variants must be present in the PPV resistant ‘Goldrich’ haplotype (454 BAC contig sequences) and be heterozygous in the diploid ‘Goldrich’ WGS; (ii) variants should be homozygous in ‘Stella’ but heterozygous in the other PPV resistant cultivars; (iii) variants must not be present in any PPV susceptible cultivar. Peach genome annotation available from the GDR was used as a reference to identify polymorphisms associated with the predicted transcripts.

### *ParPMC2* cDNA amplification and sequencing

Complete coding DNA sequences (CDS) of *ParPMC2* susceptible (S) and resistant (R) alleles were PCR-amplified with the primers pair cDNA_EcoRI_F/cDNA_BamHI_R (Additional file 14: Table S11) using ‘Goldrich’ leaf cDNA as template. This cDNA was synthesized from 500 ng of total RNA using the SuperScript III First-Strand Synthesis System kit (Invitrogen). Cycling protocol consisted of 2 min at 95 °C; 10 cycles of 30 s at 95 °C, 30 s at 49 °C (+ 0.5 °C every cycle) and 1 min at 60 °C; 25 cycles of 30 s at 95 °C, 30 s at 57 °C and 1 min (+ 10 s every cycle) at 72 °C; and finally 72° for 10 min. PCRs were performed in a final volume of 25 μL containing 10 × buffer, 1.8 mM MgCl_2_, 0.2 mM of each dNTP, 400 mM of each primer, 1 U of FastStart Taq DNA Polymerase (Roche) and 2 μL of cDNA template. PCR products were electrophoresed in 1% (*w*/*v*) agarose gels. PCR fragments were cloned into pGEM-T vector (Promega) according to the manufacturer’s instruction. DNA sequencing was performed in an ABI Prism 3130XL genetic analyzer, following manufacturer instructions for the BigDye terminator v.3.1 cycle sequencing kit (Applied Biosystems, Foster City, CA), using T7 and SP6 universal primers. Forward and reverse sequences were assembled and edited with the Staden package v.1.6-r (http://staden.sourceforge.net/). Pairwise alignment was made using the ClustalW function in BioEdit version 7.0.9 [20]. Complete CDS of *ParPMC2* resistant (R) and susceptible (S) alleles are deposited under GenBank accession numbers MF346726 and MF346727, respectively.

### *ParPMC2* allele-specific PCR assay

*ParPMC2* R- and S-alleles were specifically PCR-amplified using two forward specific primers (ParP-4_F_alleleS or ParP-4_F_alleleR), differing at the 3′ end, the reverse primer ParP-4_R (Fig. 4, Additional file 14: Table S11). PCRs were performed in a final volume of 20 μL containing 1 × DreamTaq buffer, 0.2 mM of each dNTP, 5 μM of each primer, 1 U of DreamTaq DNA polymerase (Thermo Fisher) and 100 ng of DNA. Cycling conditions were as follows: an initial denaturing of 95 °C for 5 min; 35 cycles of 95 °C for 30 s, 55 °C for 45 s and 72 °C for 45 s; and a final extension of 72 °C for 10 min. PCR products were electrophoresed in 1% (w/v) agarose gels.

### Phylogenetic analysis

CDS of the 9 *MATHd* peach genes clustered within the *PPVres* locus [58] were downloaded from the GDR database. Multiple sequence alignment using peach and apricot *MATHd* genes was performed using the ClustalW function in BioEdit version 7.0.9 [20]. Poorly aligned positions and divergent regions of the alignment were eliminated using Gblocks v.0.91b [7]. Model of nucleotide substitution comparison was performed by using the Akaike information criterion. The best-fitting evolutionary model (The Tamura 3-parameter model (T92) + G) was implemented in the Maximum Likelihood phylogenetic analysis using 500 bootstrap replications. Evolutionary divergences between sequences were estimated using the same evolutionary model and all codon positions, but removing ambiguous positions for each sequence pair. Pairwise identity matrix was also obtained. Evolutionary analyses were conducted in MEGA6 [51].

## Additional files

Additional file 1: Table S1. Summary of RNA-seq data. (PDF 73 kb)

Additional file 2: Table S2. Distribution of percent length coverage of the assembled apricot transcripts against peach annotated transcripts (Peach v.1.0). (PDF 49 kb)

Additional file 3: Data S1. Putative peach orthologs of the apricot assembled transcriptome. (TXT 2064 kb)

Additional file 4: Table S3. Blastn analysis of the Plum pox virus genome sequence (NCBI Reference Sequence: NC_001445.1) against the apricot assembled transcripts. (PDF 56 kb)

Additional file 5: Table S4. PPV abundance estimation based on RNA-seq sequences mapped against the apricot PPV assembled contig (c34934_g0_i1). (PDF 72 kb)

Additional file 6: Figure S1. Multidimensional scaling (MDS) plots of RNA-seq expression profiles showing sample and replicate relationships. Distance between each pair of samples is the leading log-fold change between them, defined as the root-mean-square of the largest 500 log2-fold changes between that pair of samples. (JPEG 1101 kb)

Additional file 7: Table S5. Similarity analysis between apricot and peach PPVres locus genes. (XLS 17 kb)

Additional file 8: Table S6. Apricot PPVres locus DEGs identified by RNA-seq data. (PDF 107 kb)

Additional file 9: Table S7. qRT-PCR analysis of PPVres locus MATHd genes showing differential expression according to RNA-seq data. (PDF 102 kb)

Additional file 10: Table S8. Summary of genome sequences used for variant calling analysis. (PDF 90 kb)

Additional file 11: Table S9. Filtered variants putatively associated with Plum pox virus (PPV) resistance. (XLS 121 kb)

Additional file 12: Table S10. Estimates of evolutionary divergence among the apricot and peach MATHd genes clustered in the PPVres locus. (PDF 87 kb)

Additional file 13: Methods S1. De Novo Transcriptome Assembly. (PDF 76 kb)

Additional file 14: Table S11. Primers used in this study for qRT-PCR and PCR-genotyping. (PDF 46 kb)

## Abbreviations

BLAST: Basic local alignment search tool
cDNA: Complementary deoxyribonucleic acid
CDS: Coding DNA sequences
CP: Coat protein
DEG: Diferentially Expressed Gene
FDR: False discovery rate
GDR: Genome Database for Rosaceae
GO: Gene Ontology
I: Inoculated
INDELS: Insertion/Deletion polymorphisms
MAS: Molecular Assisted Selection
MATHd: Meprin and TRAF-C homology domain
MDS: Multidimensional scaling plot
NI: Non-inoculated
NMD: Nonsense-Mediated Decay
Nr: NCBI non-redundant protein sequence
ParP: *P**runus* *ar**meniaca* PPVres
ParPMC: *Prunus armeniaca* PPVres MATHd-containing
PPV: Plum Pox virus
PPVres: PPV resistance locus
qRT-PCR: Quantitative real-time polymerase chain reaction
R: Resistant
RBH: Reciprocal best Blast Hit;
RNAseq: RNA sequencing
S: Susceptible
SNPs: Single Nucleotide Polymorphisms
SRA: Short Read Archive
TSA: Transcriptome Shotgun Assembly Sequence Database
WGS: Whole genome sequences
