# Supplementary material for: Resistance to Plum Pox Virus (PPV) in apricot (Prunus armeniaca L.) is associated with down-regulation of two MATHd genes
Source: BMC Plant Biol. 2018 Jan 27;18:25. doi: 10.1186/s12870-018-1237-1 (PMC5787289; doi:10.1186/s12870-018-1237-1)
Supplement: Supplementary file 1 — Summary of RNA-seq data. (PDF 73 kb) [file 12870_2018_1237_MOESM1_ESM.pdf]

Phenotype	Cultivar	PPV	Sample name	Replicate number	Sequences	Number sequences	Sequence Length	% Total Duplications	Total raw (bp)	Total cleaned (bp)	% retained
Susceptible	Canino	I	CA_I_rep1	1	raw	30853660	100	83,92	9324031000	9301457225	99,76
					cleaned	30853660	79-100	76,785			
			CA_I_rep2	2	raw	31033982	100	81,41			
					cleaned	31033982	80-100	74,805			
			CA_I_rep3	3	raw	31352668	100	82,83			
					cleaned	31352668	79-100	76,27			
		NI	CA_NI_rep1	4	raw	30747782	100	83,53	8821929200	8802588786	99,78
					cleaned	30747782	80-100	77,155			
			CA_NI_rep2	5_a	raw	8265644	100	75,56			
					cleaned	8265644	81-100	67,92			
				5_b	raw	18295646	100	87,73			
					cleaned	18295646	80-100	82,97			
			CA_NI_rep3	6	raw	30910220	100	83,74			
					cleaned	30910220	79-100	77,235			
Resistant	Goldrich	I	GO_I_rep1	7_a	raw	5107386	100	61,31	9272855600	9252249593	99,78
					cleaned	5107386	81-100	53,245			
				7_b	raw	25498252	100	80,85			
					cleaned	25498252	79-100	74,015			
			GO_I_rep2	8_a	raw	5513386	100	59,46			
					cleaned	5513386	79-100	51,31			
				8_b	raw	24760808	100	78,03			
					cleaned	24760808	77-100	70,875			
			GO_I_rep3	9	raw	31848724	100	82,28			
					cleaned	31848724	79-100	75,815			
		NI	GO_NI_rep1	10	raw	31776630	100	81,23	9404884400	9378146734	99,72
					cleaned	31776630	81-100	74,53			
			GO_NI_rep2	11	raw	30983646	100	82,28			
					cleaned	30983646	81-100	75,945			
			GO_NI_rep3	12	raw	31288568	100	81,4			
					cleaned	31288568	81-100	74,725			
	Stella	I	ST_I_rep1	13	raw	30041056	100	80,52	6069097800	6052472542	99,73
					cleaned	30041056	79-100	73,925			
			ST_I_rep2	14	raw	30649922	100	83,73			
					cleaned	30649922	80-100	78,0015			
		NI	ST-1	15	raw	31044396	100	81,73	6113926200	6098143369	99,74
					cleaned	31044396	79-100	75,265			
			ST-2	16	raw	30094866	100	83,34			
					cleaned	30094866	77-100	76,995			
TOTAL									49006724200	48885058249	99,75

Table S1. Summary of RNA-seq data. Phenotype (PPV susceptibility/resistance), Cultivar, PPV infection conditions (I: inoculated; NI: non-inoculated), Sample name, Replicate number, Sequences type (raw/cleaned), Number of sequences, Sequence length, % of total duplications, Number of raw and cleaned sequences (bp) per cultivar and condition, and % of retained sequences are indicated.
