# Supplementary material for: Resistance to Plum Pox Virus (PPV) in apricot (Prunus armeniaca L.) is associated with down-regulation of two MATHd genes
Source: BMC Plant Biol. 2018 Jan 27;18:25. doi: 10.1186/s12870-018-1237-1 (PMC5787289; doi:10.1186/s12870-018-1237-1)
Supplement: Supplementary file 2 — Distribution of percent length coverage of the assembled apricot transcripts against peach annotated transcripts (Peach v.1.0). (PDF 49 kb) [file 12870_2018_1237_MOESM2_ESM.pdf]

% coverage peach transcript	nº apricot transcripts	∑nº with ≥ coverage
100	9334	9334
90	1560	10894
80	1431	12325
70	1577	13902
60	1727	15629
50	1137	16766
40	963	17729
30	797	18526
20	864	19390
10	0	19390
0	0	19390

using BLAST+ e-value>1e-7

Table S2. Distribution of percent length coverage of the assembled apricot transcripts against peach annotated transcripts (Peach v.1.0).
