# Supplementary material for: Resistance to Plum Pox Virus (PPV) in apricot (Prunus armeniaca L.) is associated with down-regulation of two MATHd genes
Source: BMC Plant Biol. 2018 Jan 27;18:25. doi: 10.1186/s12870-018-1237-1 (PMC5787289; doi:10.1186/s12870-018-1237-1)
Supplement: Supplementary file 4 — Blastn analysis of the Plum pox virus genome sequence (NCBI Reference Sequence: NC_001445.1) against the apricot assembled transcripts. (PDF 56 kb) [file 12870_2018_1237_MOESM4_ESM.pdf]

query_id	subject_id	% identity	alignment length	mismatches	gap opens	q.start	q. end	s. start	s. end	evalue*	bit score
gi|9626508|ref|NC_001445.1|	c34934_g0_i1	98.39	8594	138	0	19	8612	1	8594	0.0	1.511e+04
gi|9626508|ref|NC_001445.1|	c34934_g0_i1	98.92	1106	12	0	8612	9717	8639	9744	0.0	1977

*using BLAST+ e-value>1e-7

Table S3. Blastn analysis of the *Plum pox virus* genome sequence (NCBI Reference Sequence: NC_001445.1) against the apricot assembled transcripts.
