# Supplementary material for: Resistance to Plum Pox Virus (PPV) in apricot (Prunus armeniaca L.) is associated with down-regulation of two MATHd genes
Source: BMC Plant Biol. 2018 Jan 27;18:25. doi: 10.1186/s12870-018-1237-1 (PMC5787289; doi:10.1186/s12870-018-1237-1)
Supplement: Supplementary file 5 — PPV abundance estimation based on RNA-seq sequences mapped against the apricot PPV assembled contig (c34934_g0_i1). (PDF 72 kb) [file 12870_2018_1237_MOESM5_ESM.pdf]

Phenotype	Sample	Counts	TPM
Susceptible	CA_I_1	3970	33.00
	CA_I_2	65597	535.96
	CA_I_3	12153	97.90
	CA_NI_1	21	0.17
	CA_NI_2	24	0.25
	CA_NI_3	17	0.14
Resistant	GO_I_1	12	0.10
	GO_I_2	28	0.25
	GO_I_3	16	0.13
	GO_NI_1	0	0.00
	GO_NI_2	1	0.01
	GO_NI_3	0	0.00
	ST_I_1	0	0.00
	ST_I_2	5	0.04
	ST_NI_1	9	0.07
	ST_NI_2	17	0.15

Table S4. PPV abundance estimation based on RNA-seq sequences mapped against the apricot PPV assembled contig (*c34934_g0_i1*). Phenotype (susceptible/resistant), Sample (Cultivars: CA (Canino), GO (Goldrich) and ST (Stella); PPV-inoculated (I) and non-inoculated (NI)), Number of reads aligning to the PPV contig and Transcripts per million (TPM) are indicated.
