# Supplementary figures and images for: Resistance to Plum Pox Virus (PPV) in apricot (Prunus armeniaca L.) is associated with down-regulation of two MATHd genes
Source: BMC Plant Biol. 2018 Jan 27;18:25. doi: 10.1186/s12870-018-1237-1 (PMC5787289; doi:10.1186/s12870-018-1237-1)

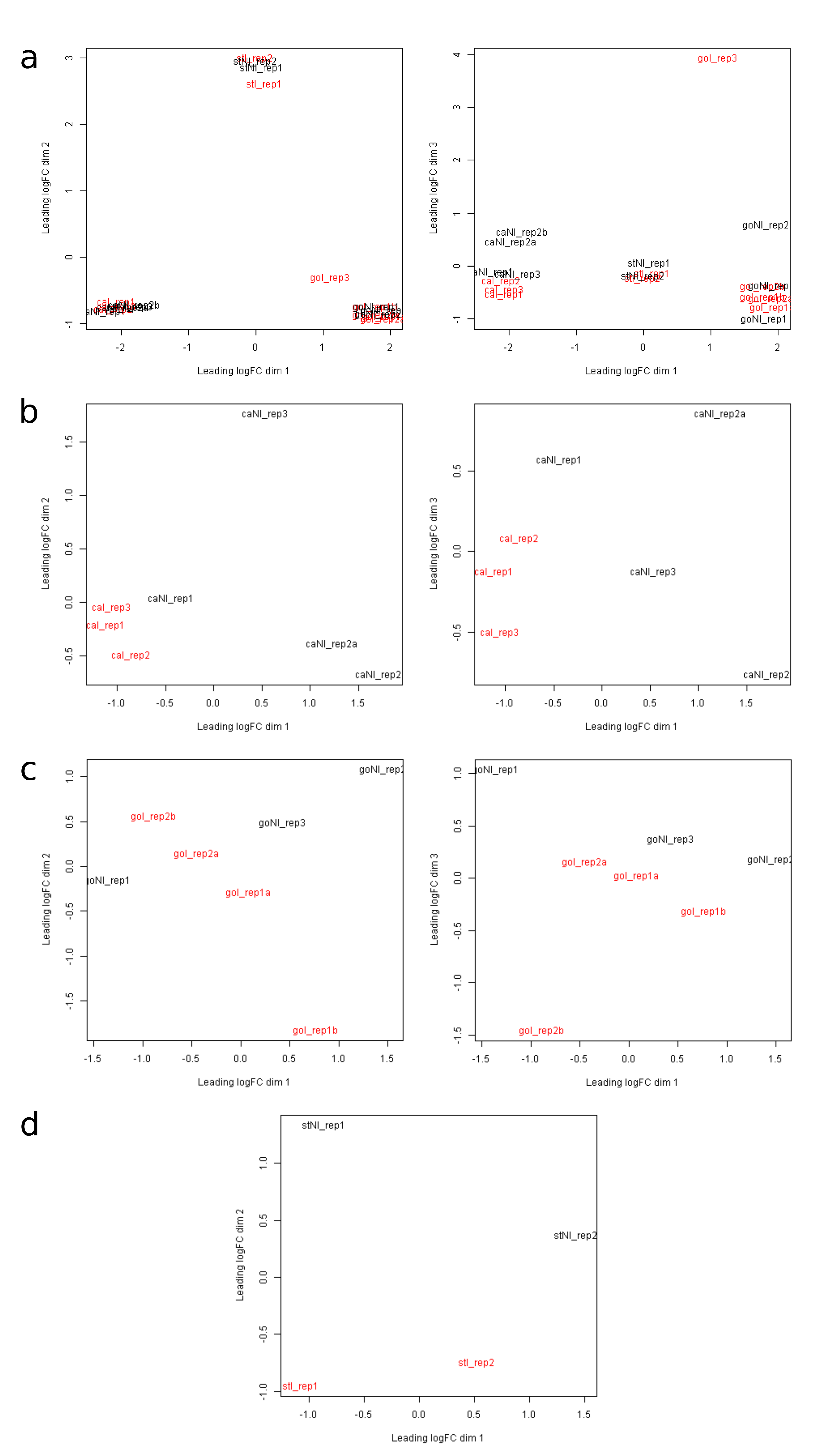

Supplement: Supplementary file 6 — Multidimensional scaling (MDS) plots of RNA-seq expression profiles showing sample and replicate relationships. Distance between each pair of samples is the leading log-fold change between them, defined as the root-mean-square of the largest 500 log2-fold changes between that pair of samples. (JPEG 1101 kb) [file 12870_2018_1237_MOESM6_ESM.jpg]
