# Supplementary material for: Resistance to Plum Pox Virus (PPV) in apricot (Prunus armeniaca L.) is associated with down-regulation of two MATHd genes
Source: BMC Plant Biol. 2018 Jan 27;18:25. doi: 10.1186/s12870-018-1237-1 (PMC5787289; doi:10.1186/s12870-018-1237-1)
Supplement: Supplementary file 8 — Apricot PPVres locus DEGs identified by RNA-seq data. (PDF 107 kb) [file 12870_2018_1237_MOESM8_ESM.pdf]

Apricot gene		Similar Peach gene	Ca vs St PPV+			Ca vs St PPV-			Ca vs Go PPV+			Ca vs Go PPV-		
Name	Trinity contig name		LogFC	logCPM	Pvalue	LogFC	logCPM	Pvalue	LogFC	logCPM	Pvalue	LogFC	logCPM	Pvalue
**ParP-1**	c37771_g2	ppa020498m (Prupe.1G106000); ppa024143m (Prupe.1G10840)	1,4498	3,3019	0,0000	1,3525	3,3960	0,0000	1,4382	3,3083	0,0000	1,2353	3,4236	0,0001
**ParP-2**	c33074_g0	ppa013518m* (Prupe.1G10710)	0,9519	4,0838	0,0000	1,4521	4,5030	0,0000	0,8407	4,0375	0,0000	0,8836	4,2302	0,0039
**ParP-3**	c36090_g2	ppa022254m* (Prupe.1G10720)	-7,0420	0,0258	0,0000	-6,3250	-0,5675	0,0003	0,1580	0,6760	0,7497[+]	0,1714	0,0778	0,8791[+]
**ParP-4**	c36090_g0	ppb022195m* (Prupe.1G10730)	-5,1987	0,5443	0,0000	-7,4080	0,3277	0,0000	-0,9907	0,8792	0,0235	-1,4559	0,4639	0,0109
**ParP-5**	c36090_g1	ppa008951m (Prupe.1G10740)	1,1054	2,1701	0,0039	1,2998	1,9399	0,0160	0,7212	1,9818	0,0632[+]	0,9938	1,8389	0,0381
**ParP-6**	c34152_g0	ppa010512m*; ppa021156m (Prupe.1G108500)	0,7753	5,0717	0,0000	0,4390	5,2026	0,1057[+]	0,5522	4,9725	0,0004	0,4638	5,2410	0,0536[+]

*RBH

[+]non-significant $p > 0.05$

Table S6. Apricot *PPVres* locus DEGs identified by RNA-seq data. Apricot gene name, homologous peach gene (v1.0 and v2.1; asterisk indicates RBH), logarithm of the fold-change (LogFC) between both samples (Cultivars: CA (Canino), ST (Stella) and GO (Goldrich); PPV-inoculated (+) and PPV-non-inoculated (-)), logarithm of the counts per million (logCPM) and the corrected p-value are indicated for each comparison.
