# Supplementary material for: Resistance to Plum Pox Virus (PPV) in apricot (Prunus armeniaca L.) is associated with down-regulation of two MATHd genes
Source: BMC Plant Biol. 2018 Jan 27;18:25. doi: 10.1186/s12870-018-1237-1 (PMC5787289; doi:10.1186/s12870-018-1237-1)
Supplement: Supplementary file 9 — qRT-PCR analysis of PPVres locus MATHd genes showing differential expression according to RNA-seq data. (PDF 102 kb) [file 12870_2018_1237_MOESM9_ESM.pdf]

			ParP-3			*ParP-4*			*ParP-5*		
**Phenotype**	**Sample**	**PPV**	**Normalized Target Value**	**SD**	**Relative Expression**	**Normalized Target Value**	**SD**	**Relative Expression**	**Normalized Target Value**	**SD**	**Relative Expression**
S	CA+	+	3,130	0,232	195,615	1,083	0,080	7,645	0,576	0,443	0,657
	CA-	-	4,844	0,325	302,714	1,263	0,102	8,913	0,647	0,541	0,738
R	GO+	+	1,104	0,124	68,974	0,445	0,048	3,137	0,592	0,577	0,675
	GO-	-	1,715	0,114	107,192	0,513	0,025	3,622	0,703	0,506	0,801
	ST+	+	0,013	0,000	0,826	0,105	0,002	0,739	0,924	0,194	1,054
	ST-	-	0,016	0,000	1,000	0,142	0,003	1,000	0,877	0,336	1,000
S	CA	-	4,844	0,325	302,714	1,263	0,102	8,913	0,647	0,541	0,738
	CU	-	28,906	1,987	1806,312	1,572	0,037	11,096	0,979	0,241	1,116
	GI	-	27,071	1,844	1691,653	1,218	0,043	8,594	0,615	0,207	0,702
	KA	-	68,289	4,176	4267,316	2,491	0,038	17,579	1,391	0,193	1,586
	MI	-	14,270	1,456	891,702	1,169	0,037	8,250	1,250	0,247	1,425
R	OR	-	0,023	0,001	1,408	0,132	0,002	0,929	0,247	0,047	0,282
	HA	-	0,014	0,000	0,874	0,419	0,002	2,959	0,673	0,096	0,768
	GO	-	1,715	0,114	107,192	0,513	0,025	3,622	0,703	0,506	0,801
	ST	-	0,016	0,000	1,000	0,142	0,003	1,000	0,877	0,336	1,000

Table S7. qRT-PCR analysis of *PPVres* locus *MATHd* genes showing differential expression according to RNA-seq data. Phenotype (R, resistant; S, susceptible), cultivar (CA, Canino; CU, Currot; GI, Ginesta; KA, Katy; MI, Mitger; OR, Orange Red; HA, Harlayne; GO, Goldrich; ST, Stella), PPV inoculation (+, inoculated; -, non-inoculated), Normalized Expression (using housekeeping genes *Actin* and *Sand-like* as controls), Standard Deviation (SD), Relative Expression against the non-inoculated ‘Stella’ sample. Data are means from 1-3 biological samples with 3 technical replicates.
