# Supplementary material for: Resistance to Plum Pox Virus (PPV) in apricot (Prunus armeniaca L.) is associated with down-regulation of two MATHd genes
Source: BMC Plant Biol. 2018 Jan 27;18:25. doi: 10.1186/s12870-018-1237-1 (PMC5787289; doi:10.1186/s12870-018-1237-1)
Supplement: Supplementary file 10 — Summary of genome sequences used for variant calling analysis. (PDF 90 kb) [file 12870_2018_1237_MOESM10_ESM.pdf]

							Mapped sequences PPVres locus		Variants PPVres locus		
**Sample**	**Phenotype**	**Phenotype Reference**	**NGS platform**	**NGS Reference**	**No. raw sequences**	**Cleaned sequences**	**Nº sequences**	**Bases mapped**	**SNPs**	**indels**	
**Bora**	**R**	Poggi Pollini et al., 2008	Illumina HiSeq 2500	Mariette et al., 2015	75344454	74811478	31075	2780581	3330	548	
**Early Blush**	**R**	Mariette et al., 2015	Illumina HiSeq 2500	Mariette et al., 2015	39650940	39195697	20158	1807143	3153	488	
**Goldrich**	**R**	Dosba et al., 1992	454	Zuriaga et al. 2013	127802	109335	19482	6667080	2424	425	
			Illumina HiSeq 2000	Zuriaga et al. 2013	137954275	130871579	56441	5139762	3570	642	
**Harcot**	**R**	Dosba et al., 1992	Illumina HiSeq 2500	Mariette et al., 2015	218574844	213377770	92471	8526586	3320	552	
**Harlayne**	**R**	Dosba et al., 1992	Illumina HiSeq 2000	Zuriaga et al. 2013	91443246	87258764	55033	5025882	3468	620	
**Henderson**	**R**	Dosba et al., 1989	Illumina HiSeq 2500	Mariette et al., 2015	58616272	56854158	26938	2453707	3163	496	
**Orange Red**	**R**	Fuchs et al. 1998	Illumina HiSeq 2500	Mariette et al., 2015	38381772	35912056	16490	1459224	3134	481	
**Stark Early Orange**	**R**	Syrgiannidis 1980	Illumina HiSeq 2000	Zuriaga et al. 2013	156657196	149154961	91759	8352167	3640	638	
**Stella**	**R**	Syrgiannidis 1980	Illumina HiSeq 2000	Mariette et al., 2015	276885698	273836121	130355	11595590	3088	541	
**Veecot**	**R**	Dosba et al., 1992	Illumina HiSeq 2500	Mariette et al., 2015	72451224	71965887	39410	3553378	3277	522	
**Arrogante**	**S**	Martínez-Gómez et al., 2000	Illumina HiSeq 2500	Mariette et al., 2015	57452476	57088462	31075	2780581	2850	490	
**Bebeco**	**S**	Dosba et al., 1989	Illumina HiSeq 2500	Mariette et al., 2015	151088024	148633539	76510	6685777	3107	516	
**Bergeron**	**S**	Karayiannis 1989	Illumina HiSeq 2500	Mariette et al., 2015	79281944	78200392	38073	3412759	3178	531	
**Bulida**	**S**	Martínez-Gómez et al., 2000	Illumina HiSeq 2500	Mariette et al., 2015	57137724	56666434	30038	2672725	3205	544	
**Canino**	**S**	Avinent et al., 1993	Illumina HiSeq 2000	Zuriaga et al. 2013	373801518[a]	129438652	77359	7166758	3600	651	
**Hargrand**	**S**	Mariette et al., 2015	Illumina HiSeq 2500	Mariette et al., 2015	42447764	41010963	18651	1675329	2801	448	
**Katy**	**S**	Evaluated at IVIA	Illumina HiSeq 2000	Muñoz-Sanz et al., in press	69042494	69020868	38912	3612669	3437	630	
**Krasnoshchekii**	**S**	Evaluated at IVIA	Illumina HiSeq 2000	Zuriaga et al. 2013	142966212	137511644	59750	5459004	3599	636	
**Lambertin**	**S**	Mariette et al., 2015	Illumina HiSeq 2500	Mariette et al., 2015	275826032	271308860	137278	12418541	3481	600	
**Moniqui**	**S**	Dosba et al., 1992	Illumina HiSeq 2500	Mariette et al., 2015	104169396	102432453	54533	4710384	3384	554	
**Perfection**	**S**	Mariette et al., 2015	Illumina HiSeq 2500	Mariette et al., 2015	167226720	165422097	83526	7593740	3404	569	
**Reale d'Imola**	**S**	Tradafirescu and Topor 1999	Illumina HiSeq 2000	Zuriaga et al. 2013	161083342	152255001	32924	2973766	3325	601	
**Shalakh**	**S**	Karayiannis 1989	Illumina HiSeq 2000	Zuriaga et al. 2013	97418198	93361180	35825	3284022	3427	608	
**Velazquez**	**S**	Martínez-Gómez et al., 2000	Illumina HiSeq 2500	Mariette et al., 2015	46142574	45802173	25552	2273819	2947	465	
**Prunus mume**	**S**	James and Thompson 2006	Illumina HiSeq 2000	Zuriaga et al. 2013	128834518	123740463	34872	3267893	3244	516	
**Prunus sibirica**	**S**	James and Thompson 2006	Illumina HiSeq 2000	Zuriaga et al. 2013	155337008	149787409	76594	7027136	3928	711	
**Global Average**					111597775	109445496,1	53003,11111	4976889	3277,19	556,41	**Global average**
					[a]Due to the high coverage, 1/3 of cleaned sequences were randomly selected				3252,48	551,84	**Apricot average**

Table S8. Summary of genome sequences used for variant calling analysis. Sample, Phenotype (R, Resistance; S, Susceptibility), Phenotype reference, NGS platform, NGS data reference, Number of raw and cleaned sequences, Number of sequences and bases mapped against the *PPVres* locus, and Number of identified Variants (SNPs and small INDELs) are indicated.
