# Supplementary material for: Resistance to Plum Pox Virus (PPV) in apricot (Prunus armeniaca L.) is associated with down-regulation of two MATHd genes
Source: BMC Plant Biol. 2018 Jan 27;18:25. doi: 10.1186/s12870-018-1237-1 (PMC5787289; doi:10.1186/s12870-018-1237-1)
Supplement: Supplementary file 12 — Estimates of evolutionary divergence among the apricot and peach MATHd genes clustered in the PPVres locus. (PDF 87 kb) [file 12870_2018_1237_MOESM12_ESM.pdf]

Distance_data_apricot_peach_aln

		Identity													
		ppa017827m	ppa024696m	c36090_g4	ppa023061m	ppa019595m	c36090_g3	ppa008960m	ppb022195m	ParPMC2	ppa008951m	ParP-5	ppa022254m	ParPMC1	ppb020867m
	ppa017827m		0,818	0,824	0,814	0,8	0,807	0,704	0,697	0,702	0,698	0,619	0,691	0,682	0,416
	ppa024696m	0,233		0,973	0,797	0,774	0,835	0,699	0,678	0,726	0,684	0,619	0,682	0,722	0,418
	c36090_g4	0,222	0,028		0,798	0,775	0,838	0,7	0,684	0,734	0,689	0,622	0,684	0,722	0,416
	ppa023061m	0,172	0,209	0,207		0,815	0,794	0,678	0,671	0,683	0,668	0,595	0,651	0,657	0,397
	ppa019595m	0,209	0,266	0,264	0,197		0,917	0,667	0,646	0,657	0,659	0,58	0,639	0,649	0,396
	c36090_g3	0,233	0,195	0,190	0,196	0,063		0,694	0,665	0,713	0,689	0,61	0,662	0,703	0,42
evolutionary divergence	ppa008960m	0,472	0,503	0,501	0,470	0,520	0,492		0,674	0,678	0,703	0,613	0,664	0,665	0,495
	ppb022195m	0,491	0,562	0,543	0,495	0,593	0,578	0,563		0,931	0,788	0,729	0,842	0,822	0,405
	ParPMC2	0,477	0,426	0,405	0,449	0,544	0,436	0,551	0,069		0,784	0,734	0,832	0,875	0,412
	ppa008951m	0,489	0,542	0,527	0,496	0,543	0,504	0,477	0,290	0,306		0,791	0,79	0,78	0,416
	ParP-5	0,763	0,778	0,765	0,759	0,858	0,785	0,785	0,420	0,419	0,292		0,769	0,747	0,432
	ppa022254m	0,506	0,542	0,537	0,545	0,608	0,585	0,589	0,190	0,215	0,292	0,330		0,895	0,406
	ParPMC1	0,533	0,430	0,432	0,521	0,572	0,460	0,587	0,222	0,148	0,312	0,382	0,122		0,414
	ppb020867m	0,837	0,851	0,865	0,857	0,857	0,798	0,451	0,905	0,849	0,822	0,727	0,879	0,827	

Table S10. Estimates of evolutionary divergence among the apricot and peach *MATHd* genes clustered in the *PPVres* locus. Confident positions (935 bases) from the alignment of peach CDS sequences and apricot assembled genes were used. Lower diagonal shows the number of base substitutions per site between sequences. The Tamura 3-parameter model (T92) + G was used as the best-fitting evolutionary model. Upper diagonal shows the percentage of base identity between sequences.
