# Supplementary material for: Resistance to Plum Pox Virus (PPV) in apricot (Prunus armeniaca L.) is associated with down-regulation of two MATHd genes
Source: BMC Plant Biol. 2018 Jan 27;18:25. doi: 10.1186/s12870-018-1237-1 (PMC5787289; doi:10.1186/s12870-018-1237-1)
Supplement: Supplementary file 13 — Methods S1. De Novo Transcriptome Assembly. (PDF 76 kb) [file 12870_2018_1237_MOESM13_ESM.pdf]

Methods S1. ***De Novo*** **Transcriptome Assembly details**

High quality reads of all samples were combined in order to perform the *de novo* assembly using the Trinity v.20140413p1 software (Grabherr *et al.*, 2011). To increase computational efficiency, repeated reads were reduced using the 'insilico_read_normalization.pl' script belonging to the Trinity software ('pair_together', 'max_cov' 30) as suggested by Haas *et al*. (2013). The assembly was sequentially refined. First, highly similar transcripts were collapsed using Cap3 software (Huang and Madan, 1999; Yang and Smith, 2013). As a result, assembled transcripts are renamed in the file with '.contigs' extension and not modified transcripts are written in the file with '.singlets' extension. Second, the 'rename_cap3_contigs_to_trinity_subcomponents.py' script available as part of seq_crumbs was employed to rename the sequences from the '.contigs' file in order to make them compatibles with the Trinity naming and the obtained file was joined with the 'singlets' file. Third, the 'filter_by_complexity' script was employed in order to eliminate low complexity transcripts. Finally, the 'trinity_split_subcomp_by_transblast.py' script was employed to analyze each cluster and split it into subcomponents if they do not share at least 100 bp and 97% similarity.

Huang, X. and Madan, A. (1999) CAP3: A DNA Sequence Assembly Program. *Genome Res.,* **9**, 868-877.

Yang, Y. and Smith, S.A. (2013) Optimizing de novo assembly of short-read RNA-seq data for phylogenomics. *BMC Genomics,* **14**, 328-328.
