# Supplementary material for: Resistance to Plum Pox Virus (PPV) in apricot (Prunus armeniaca L.) is associated with down-regulation of two MATHd genes
Source: BMC Plant Biol. 2018 Jan 27;18:25. doi: 10.1186/s12870-018-1237-1 (PMC5787289; doi:10.1186/s12870-018-1237-1)
Supplement: Supplementary file 14 — Primers used in this study for qRT-PCR and PCR-genotyping. (PDF 46 kb) [file 12870_2018_1237_MOESM14_ESM.pdf]

Gene	Primers	Sequence 5’-3’
ParP-4	F	TGCCAACTCATTACACGTTCA
	R	GTGCTCTTTCACATTCTTGCTC
	F_alleleS	GTCGTTTTCATTGATGTCCAAAC
	F_alleleR	GTCATTTTCATTGATGTCATTCA
	cDNA_EcoRI_F	GGCGAATTCATGAGCATGAATAACCTTAACTTCGA
	cDNA_BamHI_R	TTAGGATCCTTAGTCTAGCGCACTAGCAGTT
ParP-5	F	TCTTCCTCCAGGCTCTAAAATCTAT
	R	AAATCCTAGCGTTATGAATCTCCAC
ParP-3	F	AGAATACTGGCACGTACTTTGCTC
	R	GTCAGTAAAAGCTTTGAGGGAGAG
Actin	ACT3	CTTCTTACTGAGGCACCCCTGAAT
	ACT4	AGCATAGAGGGAGAGAACTGCTTG
Sand-like	F	TCGTGGGTACCAGGAAAACGACAT
	R	CCTGCTAGCTTGTGTTCATCTCCA

Table S11. Primers used in this study for qRT-PCR and PCR-genotyping.
